# The Brabazon Home of Comfort

**Published:** 1887-04-02

**Authors:** Annie Cazenove

**Affiliations:** Hon. Sec.


### The Brabazon Home of Comfort.

By Annie Cazenove, Hon. Sec.

This Home at Reigate—unique of its kind—was opened in July, 1885. Its objects are as follows: It is founded by the Lady Brabazon for the reception of members of the Girls' Friendly Society (now numbering more than 100,000 in Great Britain alone, without the Colonies and America) who, not being eligible for a Hospital or Home of Rest, would otherwise linger out their days in a Workhouse Infirmary. Invalids will also be admitted who require medical care and nursing, such as they could not have in their own homes, and who without this care would be likely to become incurable.

The history of the founding of this Home—which is meeting a great want—is as follows: A lady originated the scheme and wrote a paper, which was read at a G. F. S. meeting in London, and which met with much sympathy. Lady Brabazon—always to the fore in most good works, and especially interested in anything for the care of the sick poor—took the matter up; many kind friends came forward with money; a suitable house came into the market in Reigate, with a promise of a handsome gift of most of the furniture, and Lady Brabazon herself bought the house, letting it to a committee consisting of various G. F. S. representatives and local ladies and gentlemen, for a nominal rent. Miss C. M. Yonge named the house "The Home of Comfort," and "Brabazon" was added out of compliment to the foundress, to whom all the inmates owe so much.

Everyone knows the difficulties of getting such a Home into order; but in a very few months all was ready, and the Home opened with two or three sick girls already in. As staff we have a lady superintendent, Miss Innes (an Edinburgh nurse); a working housekeeper, who cooks and looks after the four G. F. S. members always in training for servants, who do the housework; and we also have a young woman in training for sick-nursing under Miss Innes.

The Home filled very soon, and we now refuse cases for lack of room. We have eighteen invalids and four in training. The rooms are airy and lofty; the house in an excellent position just outside Reigate town, and being near the station, doctor, church, and shops, with all the benefits of the beautiful country, is most convenient. There is a large cheerful day-room and dining-room, and four large and four small wards, with one extra room, and a capital bath-room. Dr. Stone, of Reigate, gives his services, and to his skill and kindness most of the great success of the scheme is due. The accounts are managed by the Rev. G. F. Slade, who looks over the books every week with one of the ladies on the committee. Any individual or diocese subscribing £25 can send a patient free, and a vacancy is kept. Winchester, London, Salisbury, Chichester, St. Albans, and Canterbury all now have beds. We have had girls in the Home during 1886 from fourteen dioceses. Many have left us recovered, for a *long* nursing and rest will often just save a patient from becoming incurable, when a short time of care will give little relief, and experience has proved this with us. We take no case a hospital or Convalescent Home would admit.

Our charge is 7*s.* a week for members, and 10*s.* a week for non-members (we have no beds for these latter now), 3*s.* a week for a girl in training. We do not take consumptive or mental cases, or any one subject to fits.

The Home is just not *too* large, to be a real home, and yet it is not an institution, which we particularly want to avoid. We are very grateful for any subscriptions and donations, as of course our payments do not cover our expenses. The Home is doing most excellent work, and we are trying our utmost to prevent the words *chronic*, *incurable*, and *homeless*, being applied to members of the Girls' Friendly Society in England. Those who are permanently ill, with no home, find here a peaceful asylum, where they may end their days in comfort.

We much need at present easy chairs, an air cushion, India-rubber bed-bath, India-rubber hot bottles, cushions, books, pictures, and red blankets.

All further particulars, and a copy of the report for 1886, will be gladly supplied by Miss Annie Cazenove, Betchworth, Surrey by whom subscriptions and donations will be thankfully received.

